# Low-intensity pulsed ultrasound of different intensities differently affects myocardial ischemia/reperfusion injury by modulating cardiac oxidative stress and inflammatory reaction

**DOI:** 10.3389/fimmu.2023.1248056

**Published:** 2023-09-07

**Authors:** Quan Cao, Lian Liu, Yugang Hu, Sheng Cao, Tuantuan Tan, Xin Huang, Qing Deng, Jinling Chen, Ruiqiang Guo, Qing Zhou

**Affiliations:** ^1^ Department of Nephrology, Zhongnan Hospital of Wuhan University, Wuhan, China; ^2^ Echo Lab, Department of Ultrasound Imaging, Renmin Hospital of Wuhan University, Wuhan, China; ^3^ Department of Anesthesiology, Renmin Hospital of Wuhan University, Wuhan, China

**Keywords:** low-intensity pulsed ultrasound, myocardial ischemia/reperfusion injury, inflammatory reaction, oxidative stress, cardiomyocyte apoptosis

## Abstract

**Introduction:**

The prevalence of ischemic heart disease has reached pandemic levels worldwide. Early revascularization is currently the most effective therapy for ischemic heart diseases but paradoxically induces myocardial ischemia/reperfusion (MI/R) injury. Cardiac inflammatory reaction and oxidative stress are primarily involved in the pathology of MI/R injury. Low-intensity pulsed ultrasound (LIPUS) has been demonstrated to reduce cell injury by protecting against inflammatory reaction and oxidative stress in many diseases, including cardiovascular diseases, but rarely on MI/R injury.

**Methods:**

This study was designed to clarify whether LIPUS alleviates MI/R injury by alleviating inflammatory reaction and oxidative stress. Simultaneously, we have also tried to confirm which intensity of the LIPUS might be more suitable to ameliorate the MI/R injury, as well as to clarify the signaling mechanisms. MI/R and simulated ischemia/reperfusion (SI/R) were respectively induced in Sprague Dawley rats and human pluripotent stem cell-derived cardiomyocytes (hPSC-CMs). LIPUS treatment, biochemical measurements, cell death assay, estimation of cardiac oxidative stress and inflammatory reaction, and protein detections by western blotting were performed according to the protocol.

**Results:**

In our study, both in vivo and in vitro, LIPUS of 0.1 W/cm^2^ (LIPUS_0.1_) and 0.5 W/cm^2^ (LIPUS_0.5_) make no significant difference in the cardiomyocytes under normoxic condition. Under the hypoxic condition, MI/R injury, inflammatory reaction, and oxidative stress were partially ameliorated by LIPUS_0.5_ but were significantly aggravated by LIPUS of 2.5 W/cm^2^ (LIPUS_2.5_) both in vivo and in vitro. The activation of the apoptosis signal-regulating kinase 1 (ASK1)/c-Jun N-terminal kinase (JNK) pathway in cardiomyocytes with MI/R injury was partly rectified LIPUS_0.5_ both in vivo and in vitro.

**Conclusion:**

Our study firstly demonstrated that LIPUS of different intensities differently affects MI/R injury by regulating cardiac inflammatory reaction and oxidative stress. Modulations on the ASK1/JNK pathway are the signaling mechanism by which LIPUS_0.5_ exerts cardioprotective effects. LIPUS_0.5_ is promising for clinical translation in protecting against MI/R injury. This will be great welfare for patients suffering from MI/R injury.

## Graphical Abstract

Low-intensity pulsed ultrasound (LIPUS) of 0.5 W/cm^2^ ameliorates myocardial ischemia/reperfusion (MI/R) injury by alleviating cardiac hypoxic responses, oxidative stress, and inflammatory reaction with the apoptosis signal-regulating kinase 1 (ASK1)/c-Jun N-terminal kinase (JNK) pathway involved.

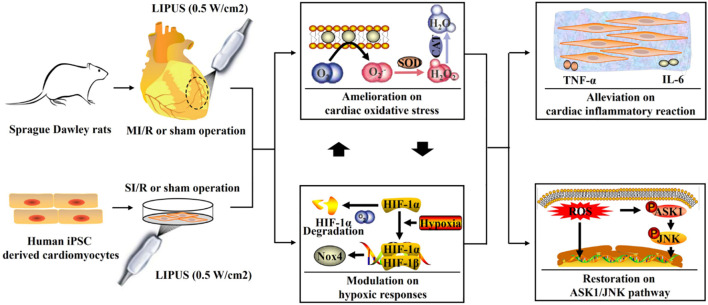

## 1 Introduction

Population aging is accelerating rapidly worldwide, the proportion of the world’s population over 60 years of age is estimated to nearly double by 2050 (1, 2). Aging increases the susceptibility to various aging-related diseases, including ischemic stroke and ischemic heart disease (1, 2). With the progress of population aging in the past few decades, the prevalence of ischemic heart disease has reached pandemic levels and has resulted in a sharp increase in mortality and morbidity worldwide (3, 4). Early revascularization is currently the most effective therapy for ischemic heart disease. Reperfusion has significantly decreased the mortality and morbidity related to ischemic heart disease (5). However, myocardial reperfusion always, paradoxically, induces and aggravates the post-ischemic injury, recognized as myocardial ischemia/reperfusion (MI/R) injury (6). Although reperfusion has been improved dramatically with the advances in revascularization agents and percutaneous coronary intervention surgery, MI/R injury is still a primary challenge for clinicians (7, 8). Therapies that aim at decreasing MI/R injury will be of great significance.

The damage inflicted on cardiomyocytes during MI/R is the consequence of two processes: ischemia per se and the subsequent reperfusion. Reperfusion to the ischemic myocardium, paradoxically, induces substantial MI/R injury owing to aggravating cardiac inflammatory reaction and oxidative stress (9). The burst of cardiac inflammatory reaction and oxidative stress greatly contribute to cardiomyocyte apoptosis, a critical pathological outcome that predicts poor prognosis (9, 10). Antioxidant and anti-inflammatory agents were thought to reduce MI/R injury theoretically but turned out to be invalid owing to the inefficiency of the reagents to enter into the cardiomyocytes (11). Besides, ischemic preconditioning is proven to decrease MI/R injury efficiently by limiting the cardiac inflammatory reaction and oxidative stress in the animal model (11, 12). However, it is unsuitable to be applied in the clinic because of potential invasive injury. Therefore, explorations of new alternative interventions for reducing MI/R injury are urgently needed.

Even though tremendous efforts have been made on reducing MI/R injury, recent therapies that can reduce MI/R injury is still far away from being satisfied (13). As a form of pulsed acoustic waves, pulsed ultrasound can transmit mechanical energy into biological tissues noninvasively (14, 15). Low-intensity pulsed ultrasound (LIPUS) (0-3 W/cm^2^) and high-intensity focused ultrasound (HIFU) (>3 W/cm^2^) have both been widely applied in the clinic in recent years (15, 16). HIFU is often applied to induce cell injury and increase cell death in thermal ablation therapy (16, 17). In recent years, few researches have studied the effects of LIPUS on different disease models with ischemia/reperfusion injury in which LIPUS has been proven to protect against cerebral ischemia/reperfusion injury and prevent recurrent ischemic stroke in the cerebral ischemia/reperfusion injury mouse model via apoptosis reduction (18, 19). Furthermore, LIPUS has been demonstrated to reduce cell injury by inhibiting inflammatory reaction and oxidative stress in many diseases, including cardiovascular diseases, but rarely on MI/R injury (20–22). Our previous study has demonstrated that LIPUS can promote cell viability and inhibits apoptosis of H9C2 cardiomyocytes in 3D bioprinting scaffolds (23). Herein, based on recent evidences, we hypothesized that LIPUS might alleviate MI/R injury by alleviating cardiac inflammatory reaction and oxidative stress. Concurrently, we also attempted to confirm which intensity of the pulsed ultrasound might be more suitable to ameliorate MI/R injury and tried to reveal the underlying mechanisms.

## 2 Materials and methods

### 2.1 Animal treatments and study design

Forty-eight Sprague Dawley rats (male, 6 weeks old, body weight 300–320 g) were purchased from Hunan SJA Laboratory Animal Co., Ltd (Changsha, Hunan, China). The rats were averagely randomized into eight groups: control rats receiving sham operations for both MI/R and LIPUS irradiation (C-sham), control rats receiving LIPUS irradiation (0.1 W/cm^2^) and sham operation for MI/R (C-LIPUS_0.1_), control rats receiving LIPUS irradiation (0.5 W/cm^2^) and sham operation for MI/R (C-LIPUS_0.5_), control rats receiving LIPUS irradiation (2.5 W/cm^2^) and sham operation for MI/R (C-LIPUS_2.5_), model rats receiving MI/R operation and sham operation for LIPUS irradiation (MI/R-sham), model rats receiving MI/R operation and LIPUS irradiation (0.1 W/cm^2^) (MI/R-LIPUS_0.1_), model rats receiving MI/R operation and LIPUS irradiation (0.5 W/cm^2^) (MI/R-LIPUS_0.5_), model rats receiving MI/R operation and LIPUS irradiation (2.5 W/cm^2^) (MI/R-LIPUS_2.5_).

Human pluripotent stem cell-derived cardiomyocytes (hPSC-CMs) were purchased from Help Stem Cell Innovations Co., Ltd (HELP4111, Nanjing, Jiangsu, China). The cardiomyocytes were averagely randomized into eight groups: control hPSC-CMs receiving sham operations for both simulated ischemia/reperfusion (SI/R) and LIPUS irradiation (C-sham), control hPSC-CMs receiving LIPUS irradiation (0.1 W/cm^2^) and sham operation for SI/R (C-LIPUS_0.1_), control hPSC-CMs receiving LIPUS irradiation (0.5 W/cm^2^) and sham operation for SI/R (C-LIPUS_0.5_), control hPSC-CMs receiving LIPUS irradiation (2.5 W/cm^2^) and sham operation for SI/R (C-LIPUS_2.5_), model hPSC-CMs receiving SI/R and sham operation for LIPUS irradiation (SI/R-sham), model hPSC-CMs receiving SI/R operation and LIPUS irradiation (0.1 W/cm^2^) (SI/R-LIPUS_0.1_), model hPSC-CMs receiving SI/R operation and LIPUS irradiation (0.5 W/cm^2^) (SI/R-LIPUS_0.5_), model hPSC-CMs receiving SI/R operation and LIPUS irradiation (2.5 W/cm^2^) (SI/R-LIPUS_2.5_).

All the rats and the hPSC-CMs were adaptively fed or cultured for three days after the randomization. All the rats were housed in a breeding facility at humidity (45-50%) and temperature (22 ± 2˚C), with unrestricted access to food and water in 12-h light/dark cycles. All the hPSC-CMs were cultured in a normoxic incubator at 20–21% O_2_. All procedures were performed according to “the National Institutes of Health guide for the care and use of Laboratory Animals (NIH Publications No. 8023, revised 1978)”. The study was approved by the Institutional Animal Care and Use Committee at Renmin Hospital, Wuhan University, China (IACUC issue no. WDRM20190614).

### 2.2 MI/R and SI/R protocol

MI/R protocol was implemented to the model rats as previously described (24). Left lateral thoracotomy was performed under inhalation anesthesia (2.0%-3.0% isoflurane, 1.0–2.0 L/min oxygen). The heart was exposed and a 6-0 suture was placed around the left anterior descending (LAD) of the left coronary artery. A ligation was induced by tightening the suture with a loop system. Regional ischemia was confirmed by the blanching of the myocardium and the elevation of ST on the ECG (LabChart, AD Instruments, Colorado Springs, USA). Following 30 mins of ischemia, the myocardium was reperfused by loosening the slipknot. The control rats underwent the same procedures but without the ligation of the LAD artery.

SI/R was performed on the model hPSC-CMs as the previous study described (25). Briefly, the hPSC-CMs were first washed twice with PBS and then switched to low-glucose serum-free DMEM. The cultures were then placed in a hypoxia chamber (Billups Rothenberg) (1%-2% O_2_, 95% N_2_, and 5% CO_2_) and maintained at 37˚C for 3h. After hypoxia, the model hPSC-CMs were reperfused by switching back to the regular culture media and placing them in a normoxic incubator with 20–21% O_2_. The control hPSC-CMs underwent the same procedures, but without culturing in the hypoxia chamber.

### 2.3 LIPUS treatment

Following the MI/R and SI/R, LIPUS (ultrasound device, Chongqing Haifu Medical Technology Co., Ltd., Chongqing, China) irradiation was performed according to the study protocol. The irradiation conditions of the LIPUS were conducted based on previous research (26). The frequency is at 1.0 MHz; repeat frequency at 100 Hz; duty cycle at 20%. The intensity of 0.1, 0.5, and 2.5 W/cm^2^ were respectively performed on the corresponding rats and hPSC-CMs according to the study design. For the rats, LIPUS was applied to the lateral wall (ischemia-reperfusion region) by a 2-dimensional scan at the parasternal long-axis view. For the hPSC-CMs, LIPUS was applied to the baseplate of the cultures by using ultrasonic coupling gel. The sham operation underwent the same procedures but without the LIPUS delivered by the ultrasound device. LIPUS irradiation was given on Days 1, 3, and 5 (20 mins a day) after MI/R and SI/R. All the rats and the hPSC-CMs were followed up in the following 14 days after MI/R.

### 2.4 Biochemical measurements

Blood samples were taken from the inferior vena cava following an overnight fast. LV samples were collected from the myocardium within the ischemic border zone in the model rats or within the corresponding zone in the control groups. The hPSC-CMs and culture medium were centrifugated (1500 rpm for 10 minutes) and collected for the following detections. Injury biomarkers in the plasma of the rats and the culture medium of the hPSC-CMs, including cardiac troponin I (cTnI), cardiac troponin T (cTnT), creatine kinase-MB (CK-MB), myoglobin, and lactate dehydrogenase (LDH), were detected for the evaluation of myocardial injury. The levels of tumor necrosis factor α (TNF-α) and interleukin-6 (IL-6) in the plasma of the rats and the culture medium of the hPSC-CMs, as well as the levels of monocyte chemoattractant protein-1 (MCP-1) and caspase-3 (Casp-3) in the myocardium, were detected to evaluate the cardiac inflammation. Protein levels of hypoxia-inducible factor 1α (HIF-1α) and NADPH oxidase 4 (NOX4) in cardiomyocytes were measured to estimate the HIF-1α-mediated hypoxic responses. The level of malondialdehyde (MDA), as well as the activity of superoxide dismutase (SOD) and catalase (CAT) in cardiomyocytes, was determined to assess cardiac oxidative stress. All the assays were conducted using corresponding assay kits according to the manufacturer’s protocol. Kits for cTnI, cTnT, CK-MB, myoglobin, IL-6, TNF-α, MCP-1, and HIF-1α were purchased from CUSABIO, Wuhan, China. LDH, NOX4, and Casp-3 were purchased from CLOUD-CLONE CORP, Wuhan, China. MDA, SOD, and CAT were purchased from Nanjing Jiancheng Bioengineering Institute, Nanjing, China.

### 2.5 Cell death assay

Myocardial apoptosis in LV samples was determined by TUNEL assays according to the manufacturer’s instructions (11684817910; Roche Diagnostics, Basel, Switzerland). The slides of LV samples were first incubated with terminal deoxynucleotidyl transferase for 60 mins at 37˚C. Diaminobenzidine was then added to the slides for 30 mins. The slides were subsequently incubated by the substrate solution for 15 mins at 37˚C. After deparaffinization and rehydration, the slides were immersed in the TUNEL reaction mixture for 1 h at 37˚C under a humidified atmosphere in the dark. Cardiomyocyte apoptosis index (%) is defined as the percentage of the TUNEL-positive cells (apoptotic cardiomyocytes had deep-brown nuclei while normal cardiomyocytes had blue nuclei) among the total number of cells under a light microscope. A total of three visual fields (Magnification: 400×) were randomly selected per rat for the analysis of myocardial apoptosis.

The hPSC-CMs were directly observed under digital light microscopy (Eclipse Ti-SR; Nikon Corporation, Tokyo, Japan). Cell viability of the hPSC-CMs was determined by cell counting kit-8 (CCK-8, Dojindo, Kumamoto, Kyushu, Japan) according to the manufacturer’s instructions. The cardiomyocytes were cultured in 24-well plates. The CCK-8 solution was added to each well and incubated with the hPSC-CMs at 37˚C for 3 h. The absorbance at 450 nm was measured using a microplate reader. The average absorbance of 6 wells was used to calculate the percentage of cell viability in each group.

### 2.6 Estimation of reactive oxygen species

Dihydroethidium staining was used to determine the ROS generation in LV samples according to the instructions of commercial kits (DHE, D7008, Sigma-Aldrich, Darmstadt, Germany). Firstly, the cryosections (10-μm) of LV samples were incubated with dihydroethidium at 37˚C for 30 mins. The ethidium fluorescence (excitation/emission at 488/610 nm) was then determined by digital fluorescence microscopy (Eclipse Ti-SR; Nikon Corporation, Tokyo, Japan). A total of three visual fields (Magnification: 400×) were randomly selected per rat for the analysis of ROS production. Image-Pro Plus 6.0 software (Media Cybernetics, Inc.) was used to analyze the relative fluorescence intensity. Relative ROS generation (fold change) was calculated by normalizing it to the C-sham group.

ROS generation in the hPSC-CMs was measured by flow cytometry analysis as previously described (27). Flow cytometer evaluation was performed using the FACSCalibur instrument (BD Biosciences, USA). The hPSC-CMs were incubated with fluorescein isothiocyanate (FITC, 1mg/ml) for 1 h at 37°C. Cellular fluorescence was measured on the FL1/FITC channel (515–545 nm). The data obtained from the cell population were analyzed using the CellQuest Pro software (BD Biosciences, USA). Relative ROS generation (fold change) was calculated by normalizing it to the C-sham group.

### 2.7 Protein detections by western blotting

Western blotting was conducted using extracts from the LV samples and hPSC-CMs. Proteins (50 μg) were electrophoresed by sodium dodecyl sulfate-polyacrylamide gel and then were transferred to a polyvinylidene difluoride membrane. The blots were incubated with corresponding primary antibodies overnight at 4°C. The membrane was then incubated with horseradish peroxidase-labeled secondary antibody IgM at room temperature for 1 h. At last, the membrane was exposed to X-ray film for the quantitative analysis of the target proteins. The expression of the target proteins was normalized to GAPDH and was expressed as fold change relative to that of the C-sham group. Primary antibodies against apoptosis signal-regulating kinase 1 (ASK1, 8662, CST, Danvers, USA), phospho-ASK1 (Ser967) (p-ASK1, 3764, CST), c-Jun N-terminal kinases (JNK, 9252, CST), phospho−JNK (Thr183+Tyr185) (p-JNK, 4668, CST) were used for the western blotting in this study.

### 2.8 Prediction of functional associations between proteins

Based on recent studies (28–31), TNF-α (Gene name: TNF), IL-6 (Gene name: IL6), MCP-1 (Gene name: Ccl2), and Casp-3 (Gene name: Casp3) are the main proteins involved in inflammatory reactions and the extrinsic apoptotic pathway while HIF-1α (Gene name: HIF1a), NOX4 (Gene name: NOX4), SOD1 (Gene name: SOD1), and CAT (Gene name: Cat) are the main proteins implicated in oxidative stress and the intrinsic apoptotic pathway. ASK1 (Gene name: Map3k5) and JNK (Gene name: Mapk8) pathways are proven to regulate apoptotic pathways with the involvement of inflammatory reaction and oxidative stress. The gene names of TNF-α, IL-6, MCP-1, Casp-3, HIF-1α, NOX4, SOD1, CAT ASK1, and JNK were uploaded into the STRING database (Version 11.5) to get protein–protein networks among these proteins for Rattus norvegicus and Homo sapiens according to the database’s instructions.

### 2.9 Statistical analysis

The results were expressed as the mean ± standard error of the mean (SEM). The homogeneity of variance was analyzed by Leven’s test. Multi−group comparisons were analyzed by one-way analysis of variance (ANOVA), followed by Tukey’s *post-hoc* test. Statistical significance was considered at P < 0.05. SPSS 22.0 was used for the analysis.

## 3 Results

### 3.1 Different intensities of LIPUS differently affects the injury and death of cardiomyocytes under the normoxic and hypoxic conditions

**Figure 1:**
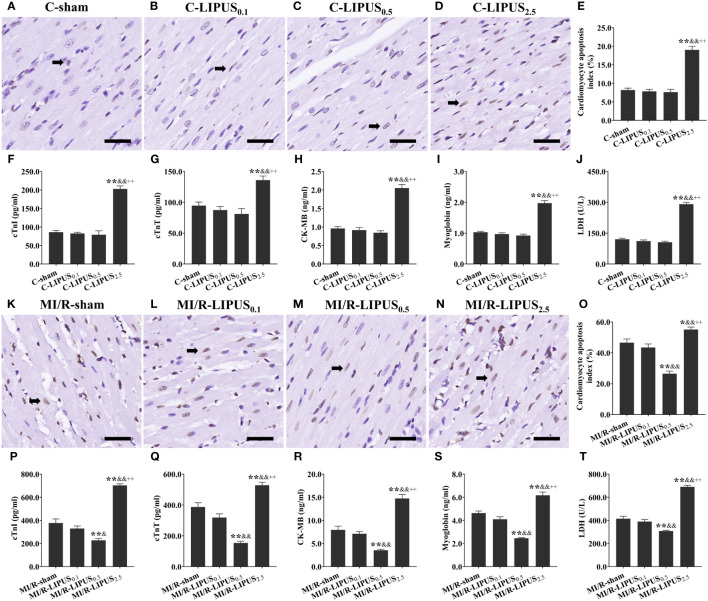
Different intensities of low-intensity pulsed ultrasound (LIPUS) differently affact myocardial injury and apoptosis in the rats without or with myocardial ischemia/reperfusion (MI/R). Representative images for the TUNEL staining of the myocardium **(A-D, K-N)**. Quantitative analysis for the effects of LIPUS on the myocardial apoptosis (black arrowheads) **(E, O)**. Effects of LIPUS on plasma injury biomarkers, including cardiac troponin I (cTnI) **(F, P)**, cardiac troponin T (cTnT) **(G, Q)**, creatine kinase MB (CK-MB) **(H, R)**, myoglobin **(I, S)**, lactate dehydrogenase (LDH) **(J, T)**. Magnification: 400×. Scale bar = 100 μm. All data are expressed as the mean ± SEM, N = 6 for each group. For the rat groups without MI/R, *: vs. C-sham group, P < 0.05; **: vs. C-sham group, P < 0.01; &: vs. C-LIPUS_0.1_, P < 0.05; &&: vs. C-LIPUS_0.1_, P < 0.01; +: vs. C-LIPUS_0.5_, P < 0.05; ++: vs. C-LIPUS_0.5_, P < 0.01. For the rat groups with MI/R, *: vs. MI/R-sham group, P < 0.05; **: vs. MI/R-sham group, P < 0.01; &: vs. MI/R-LIPUS_0.1_, P < 0.05; &&: vs. MI/R-LIPUS_0.1_, P < 0.01; +: vs. MI/R-LIPUS_0.5_, P < 0.05; ++: vs. MI/R-LIPUS_0.5_, P < 0.01.

**Figure 2:**
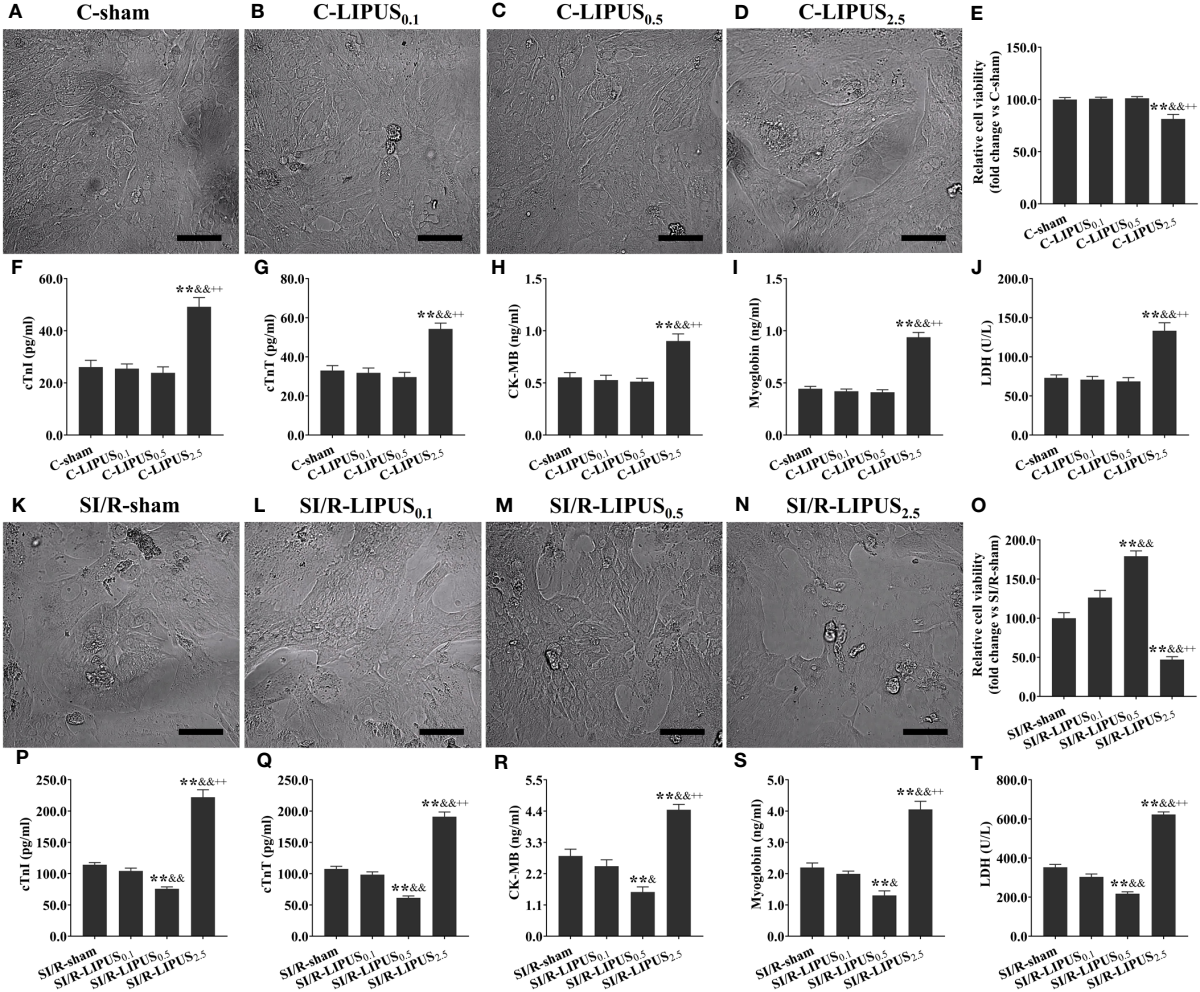
Different intensities of low-intensity pulsed ultrasound (LIPUS) exert different effects on the cell injury and viability of the human pluripotent stem cell-derived cardiomyocytes (hPSC-CMs) without or with simulated ischemia/reperfusion (SI/R). Representative images for the direct observation of the hPSC-CMs under a light microscope **(A-D, K-N)**. Effects of LIPUS on cell viability of the hPSC-CMs detected by cell counting kit-8 **(E, O)**. Effects of LIPUS on the injury biomarkers in the culture medium of the hPSC-CMs, including cardiac troponin I (cTnI) **(F, P)**, cardiac troponin T (cTnT) **(G, Q)**, creatine kinase MB (CK-MB) **(H, R)**, myoglobin **(I, S)**, lactate dehydrogenase (LDH) **(J, T)**. Magnification: 400×. Scale bar = 100 μm. All data are expressed as the mean ± SEM, N = 6 for each group. For the hPSC-CMs groups without SI/R, *: vs. C-sham group, P < 0.05; **: vs. C-sham group, P < 0.01; &: vs. C-LIPUS_0.1_, P < 0.05; &&: vs. C-LIPUS_0.1_, P < 0.01; +: vs. C-LIPUS_0.5_, P < 0.05; ++: vs. C-LIPUS_0.5_, P < 0.01. For the hPSC-CMs groups with SI/R, *: vs. SI/R-sham group, P < 0.05; **: vs. SI/R-sham group, P < 0.01; &: vs. SI/R-LIPUS_0.1_, P < 0.05; &&: vs. SI/R-LIPUS_0.1_, P < 0.01; +: vs. SI/R-LIPUS_0.5_, P < 0.05; ++: vs. SI/R-LIPUS_0.5_, P < 0.01.

Under the normoxic condition both *in vivo* and *in vitro*, the injury biomarkers (**Figures 1F–J**, **2F–J**) were not significantly different in the C-LIPUS_0.1_ and C-LIPUS_0.5_ group compared to the C-sham group but were significantly higher in the C-LIPUS_2.5_ group compared to the C-sham group; Indicated by the TUNEL staining and CCK-8 kit assay, cardiomyocyte death (**Figures 1A–E**, **2A–E**) was not significantly different among the C-sham, C-LIPUS_0.1_, and C-LIPUS_0.5_ group but was significantly increased in the C-LIPUS_2.5_ group compared to the C-sham group both *in vivo* and *in vitro* under the normoxic condition; As shown in the online **Supplementary Videos**, consistent with cell viability evaluated by the CCK-8 kit assay, cell viability of the hPSC-CMs indicated by its synchronous pulsation showed no difference among the C-sham (**Video S1** C-sham), C-LIPUS_0.1_ (**Video S2** C-LIPUS_0.1_), and C-LIPUS_0.5_ group (**Video S3** C-LIPUS_0.5_); The hPSC-CMs showed decreased cell viability in the C-LIPUS_2.5_ group (**Video S4** C-LIPUS_2.5_) compared to the C-sham group.

Under the hypoxic condition both *in vivo* and *in vitro*, the injury (**Figures 1P–T**, **2P–T**) and death (**Figures 1K–O**, **2K–O**) of the cardiomyocyte were not significantly different between the MI/R-sham (SI/R-sham) and MI/R-LIPUS_0.1_ (SI/R-LIPUS_0.1_) group; The injury and death of the cardiomyocyte was partly ameliorated in the MI/R-LIPUS_0.5_ (SI/R-LIPUS_0.5_) group compared to MI/R-sham (SI/R-sham) group; The injury and death of the cardiomyocyte were significantly aggravated in the MI/R-LIPUS_2.5_ (SI/R-LIPUS_2.5_) group compared to the MI/R-sham (SI/R-sham group) group; As shown in the online **Supplementary Videos**, consistent with cell viability evaluated by the CCK-8 kit assay, the cell viability of the hPSC-CMs indicated by its synchronous pulsation showed no difference between the SI/R-sham **Video S5** SIR-sham) and SI/R-LIPUS_0.1_ group (**Video S6** SIR-LIPUS_0.1_); The hPSC-CMs showed decreased cell viability in the SI/R-LIPUS_2.5_ (**Video S8** SIR-LIPUS_2.5_) compared to the SI/R-sham (**Video S5** SI/R-sham); The cell viability was partly ameliorated in the SI/R-LIPUS_0.5_ group (**Video S7** SIR-LIPUS_0.5_) compared to the SI/R-sham group (**Video S5** SIR-sham).

### 3.2 Protein-protein networks indicated by STRING database

**Figure 3:**
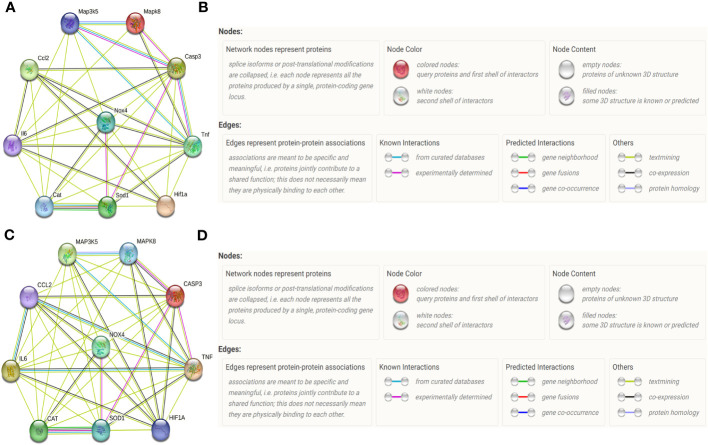
Protein-protein networks indicated by STRING database. Protein–protein networks among IL-6 (Gene name: IL6), MCP-1 (Gene name: Ccl2), Caspase-3 (Gene name: Casp3), TNF-α (Gene name: TNF), HIF-1α (Gene name: HIF1a), NOX4 (Gene name: NOX4), SOD (Gene name: SOD1), CAT (Gene name: Cat), ASK1 (Gene name: Map3k5) and JNK (Gene name: Mapk8) were indicated by STRING database for Rattus norvegicus **(A)** and the corresponding Legends **(B)**. Protein–protein networks among TNF-α, IL-6, MCP-1, Caspase-3, HIF-1α, NOX4, SOD1, CAT ASK1, and JNK were indicated by STRING database for Homo sapiens **(C)** and the corresponding Legends **(D)**.

For Rattus norvegicus (**Figures 3A, B**), recent known interactions between TNF-α and Casp3, between Casp3 and ASK1, and between CAT and Sod1, are confirmed by data from curated databases and experimentally determined; Recent known interactions between JNK and Casp3, between JNK and ASK1, between Casp3 and SOD1, between NOX4 and SOD1, are confirmed by data from curated databases or experimentally determined; Interactions with gene neighborhood are predicted between CAT and Sod1; Interactions with co-expression are predicted among most of the proteins included in our vivo experiment.

For Homo sapiens (**Figures 3C, D**), recent known interactions between CAT and Sod1is confirmed by data from curated databases and experimentally determined; Recent known interactions between Mapk8 and Casp3, between JNK and ASK1, between ASK1 and TNF-α, between TNF-α and MCP-1, between IL6 and MCP-1, between TNF and IL-6, between Mapk8 and Casp3, between TNF and Casp3, between SOD1 and Casp3, between NOX4 and SOD1 are confirmed by data from curated databases or experimentally determined; Interactions with gene neighborhood are predicted between CAT and SOD1; Interactions with co-expression are predicted among most of the proteins included in our vitro experiment.

### 3.3 Different intensities of LIPUS exert different effects on cardiac inflammation under the normoxic and hypoxic conditions

**Figure 4:**
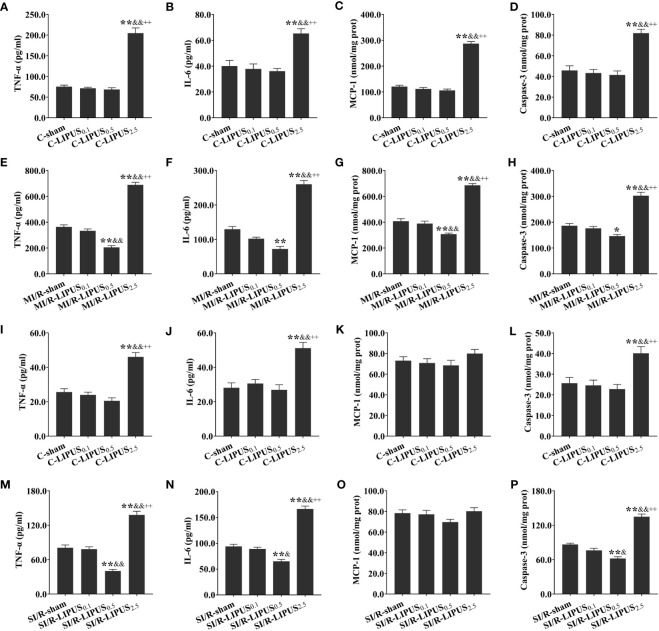
Different intensities of low-intensity pulsed ultrasound (LIPUS) exert different effects on cardiac inflammation in rats and human pluripotent stem cell-derived cardiomyocytes (hPSC-CMs) without or with myocardial ischemia/reperfusion (or simulated ischemia/reperfusion). Quantitative analysis for the effects of LIPUS on the plasma levels of tumor necrosis factor α (TNF-α) **(A, E, I, M)** and interleukin-6 (IL-6) **(B, F, J, N)**, monocyte chemoattractant protein-1 (MCP-1) **(C, G, K, O)**, Caspase-3 **(D, H, L, P)** and in the rats and hPSC-CMs. All data are expressed as the mean ± SEM, N = 6 for each group. For the rat groups without MI/R, *: vs. C-sham group, P < 0.05; **: vs. C-sham group, P < 0.01; &: vs. C-LIPUS_0.1_, P < 0.05; &&: vs. C-LIPUS_0.1_, P < 0.01; +: vs. C-LIPUS_0.5_, P < 0.05; ++: vs. C-LIPUS_0.5_, P < 0.01. For the rat groups with MI/R, *: vs. MI/R-sham group, P < 0.05; **: vs. MI/R-sham group, P < 0.01; &: vs. MI/R-LIPUS_0.1_, P < 0.05; &&: vs. MI/R-LIPUS_0.1_, P < 0.01; +: vs. MI/R-LIPUS_0.5_, P < 0.05; ++: vs. MI/R-LIPUS_0.5_, P < 0.01. For the hPSC-CMs groups without SI/R, *: vs. C-sham group, P < 0.05; **: vs. C-sham group, P < 0.01; &: vs. C-LIPUS_0.1_, P < 0.05; &&: vs. C-LIPUS_0.1_, P < 0.01; +: vs. C-LIPUS_0.5_, P < 0.05; ++: vs. C-LIPUS_0.5_, P < 0.01. For the hPSC-CMs groups with SI/R, *: vs. SI/R-sham group, P < 0.05; **: vs. SI/R-sham group, P < 0.01; &: vs. SI/R-LIPUS_0.1_, P < 0.05; &&: vs. SI/R-LIPUS_0.1_, P < 0.01; +: vs. SI/R-LIPUS_0.5_, P < 0.05; ++: vs. SI/R-LIPUS_0.5_, P < 0.01.

Under the normoxic condition both *in vivo* and *in vitro*, the levels of TNF-α (**Figures 4A, I**), IL-6 (**Figures 4B, J**), and Casp-3 (**Figures 4D, L**) were not significantly different among the C-sham, C-LIPUS_0.1_, and C-LIPUS_0.5_ group but were significantly increased in the C-LIPUS_2.5_ group compared to the C-sham group; The levels of MCP-1 (**Figures 4C, K**) were not significantly different among the C-sham, C-LIPUS_0.1_, and C-LIPUS_0.5_ group both in our vivo and vitro experiment, as well as in the C-LIPUS_2.5_ group compared to the C-sham group in our vitro experiment, but were significantly elevated in the C-LIPUS_2.5_ group compared to the C-sham group both in our vivo experiment.

Under the hypoxic condition both *in vivo* and *in vitro*, the levels of TNF-α (**Figures 4E, M**), IL-6 (**Figures 4F, N**), and Casp-3 (**Figures 4H, P**) were not significantly different between the MI/R-sham (SI/R-sham) and MI/R-LIPUS_0.1_ (SI/R-LIPUS_0.1_) group; The levels of TNF-α, IL-6, and Casp-3 were all significantly alleviated in the MI/R-LIPUS_0.5_ (SI/R-LIPUS_0.5_) group compared to the MI/R-sham (SI/R-sham) group, but were aggravated in the MI/R-LIPUS_2.5_ (SI/R-LIPUS_2.5_) group compared to the MI/R-sham (SI/R-sham) group; The levels of MCP-1 were not significantly different between MI/R-sham and MI/R-LIPUS_0.1_, but were significantly alleviated in the MI/R-LIPUS_0.5_ group compared to the MI/R-sham group, and were aggravated in the MI/R-LIPUS_2.5_ group compared to the MI/R-sham group; The levels of MCP-1 were not significantly different among the SI/R-sham, SI/R-LIPUS_0.1_, SI/R-LIPUS_0.5_, and SI/R-LIPUS_2.5_ group (**Figures 4G, O**).

### 3.4 Different intensities of LIPUS differently affect cardiac hypoxic responses and oxidative stress under the normoxic and hypoxic conditions

**Figure 5:**
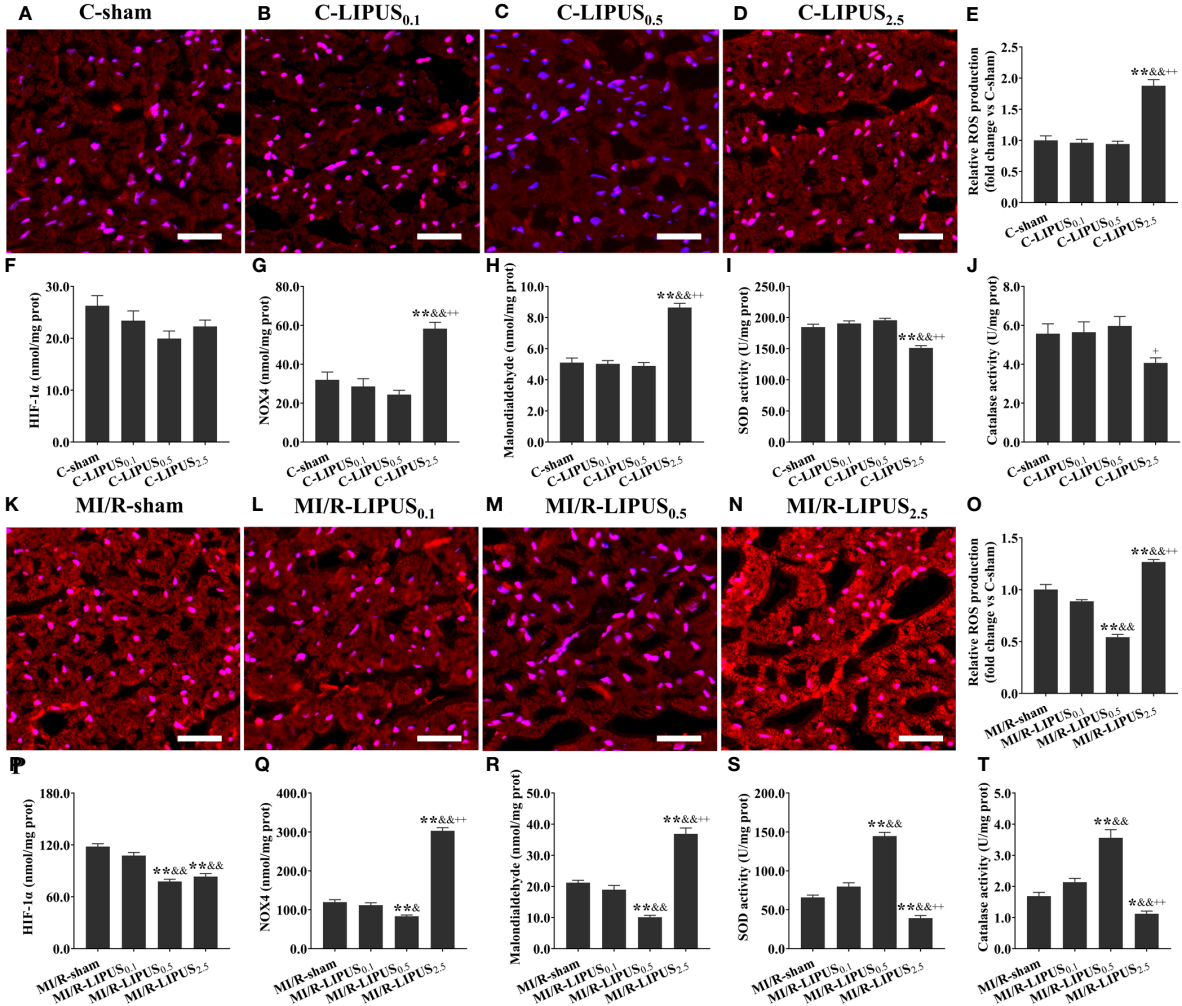
Different intensities of low-intensity pulsed ultrasound (LIPUS) differently affect cardiac hypoxic responses and oxidative stress in the rats without or with myocardial ischemia/reperfusion. Representative images for reactive oxygen species (ROS) generation determined by dihydroethidium staining in the cardiomyocytes of the rats **(A-D, K-N)**. Quantitative analysis for the effects of LIPUS on the levels of ROS **(E, O)**, hypoxia-inducible factor-1alpha (HIF-1α) **(F, P)**, NADPH oxidase 4 (Nox4) **(G, Q)**, malondialdehyde (MDA) **(H, R)**, and on the activity of superoxide dismutase (SOD) **(I, S)** and catalase (CAT) **(J, T)** in the cardiomyocytes of the rats. Magnification: 400×. Scale bar = 100 μm. All data are expressed as the mean ± SEM, N = 6 for each group. For the rat groups without MI/R, *: vs. C-sham group, P < 0.05; **: vs. C-sham group, P < 0.01; &: vs. C-LIPUS_0.1_, P < 0.05; &&: vs. C-LIPUS_0.1_, P < 0.01; +: vs. C-LIPUS_0.5_, P < 0.05; ++: vs. C-LIPUS_0.5_, P < 0.01. For the rat groups with MI/R, *: vs. MI/R-sham group, P < 0.05; **: vs. MI/R-sham group, P < 0.01; &: vs. MI/R-LIPUS_0.1_, P < 0.05; &&: vs. MI/R-LIPUS_0.1_, P < 0.01; +: vs. MI/R-LIPUS_0.5_, P < 0.05; ++: vs. MI/R-LIPUS_0.5_, P < 0.01.

**Figure 6:**
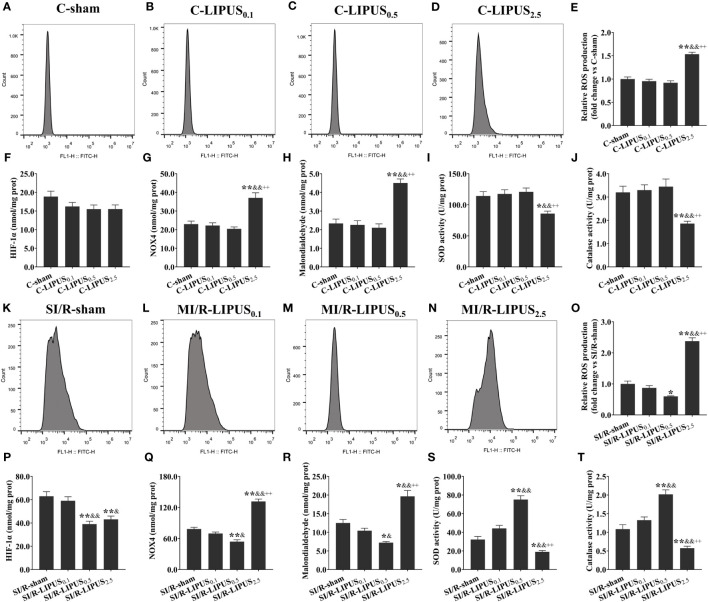
Different intensities of low-intensity pulsed ultrasound (LIPUS) differently affect cardiac hypoxic responses and oxidative stress in human pluripotent stem cell-derived cardiomyocytes (hPSC-CMs) without or with stimulated ischemia/reperfusion (SI/R). Representative images for reactive oxygen species (ROS) generation measured by flow cytometry analysis for the hPSC-CMs **(A-D, K-N)**. Quantitative analysis for the effects of LIPUS on the levels of ROS **(E, O)**, hypoxia-inducible factor-1alpha (HIF-1α) **(F, P)**, NADPH oxidase 4 (Nox4) **(G, Q)**, malondialdehyde (MDA) **(H, R)**, and on the activity of superoxide dismutase (SOD) **(I, S)** and catalase (CAT) **(J, T)** for the hPSC-CMs. Magnification: 400×. Scale bar = 100 μm. All data are expressed as the mean ± SEM, N = 6 for each group. For the hPSC-CMs groups without SI/R, *: vs. C-sham group, P < 0.05; **: vs. C-sham group, P < 0.01; &: vs. C-LIPUS_0.1_, P < 0.05; &&: vs. C-LIPUS_0.1_, P < 0.01; +: vs. C-LIPUS_0.5_, P < 0.05; ++: vs. C-LIPUS_0.5_, P < 0.01. For the hPSC-CMs groups with SI/R, *: vs. SI/R-sham group, P < 0.05; **: vs. SI/R-sham group, P < 0.01; &: vs. SI/R-LIPUS_0.1_, P < 0.05; &&: vs. SI/R-LIPUS_0.1_, P < 0.01; +: vs. SI/R-LIPUS_0.5_, P < 0.05; ++: vs. SI/R-LIPUS_0.5_, P < 0.01.

Under the normoxic condition both *in vivo* and *in vitro*, the levels of ROS (**Figures 5A-E**, **6A–E**), NOX4 (**Figures 5G**, **6G**), and MDA (**Figures 5H**, **6H**), and the activity of SOD (**Figures 5I**, **6I**) and CAT (**Figures 5J**, **6J**) in cardiomyocytes were not significantly different among the C-sham, C-LIPUS_0.1_, and C-LIPUS_0.5_ group; The levels of ROS, NOX4, and MDA increased significantly and the activity of SOD decreased obviously in the C-LIPUS_2.5_ group compared to the C-sham group; The activity of CAT showed no significant difference among C-sham, C-LIPUS_0.1_, C-LIPUS_0.5_ group, but showed a significant decrease in the C-LIPUS_2.5_ group compared to the C-LIPUS_0.1_; The levels of HIF-1a (**Figures 5F**, **6F**) were not significantly different among C-sham, C-LIPUS_0.1_, C-LIPUS_0.5_, and C-LIPUS_2.5_ group.

Under the hypoxic condition both *in vivo* and *in vitro*, the levels of ROS (**Figures 5K–O**, **6K–O**), NOX4 (**Figures 5Q**, **6Q**) and MDA (**Figures 5R**, **6R**), as well as the activity of SOD (**Figures 5S**, **6S**) and CAT (**Figures 5T**, **6T**), in cardiomyocytes were not significantly different between the MI/R-sham (SI/R-sham) and MI/R-LIPUS_0.1_ (SI/R-LIPUS_0.1_), but were significantly mitigated in MI/R-LIPUS_0.5_ (SI/R-LIPUS_0.5_) group compared to MI/R-sham (SI/R-sham), and were significantly mitigated deteriorated in MI/R-LIPUS_2.5_ (SI/R-LIPUS_2.5_) group compared to MI/R-sham (SI/R-sham); The levels of HIF-1a (**Figures 5P**, **6P**) were not significantly different between MI/R-sham (SI/R-sham) and MI/R-LIPUS_0.1_ (SI/R-LIPUS_0.1_) group, as well as between MI/R-LIPUS_0.5_ (SI/R-LIPUS_0.5_) and MI/R-LIPUS_2.5_ (SI/R-LIPUS_2.5_) group, but were significantly elevated in MI/R-LIPUS_0.5_ (SI/R-LIPUS_0.5_) and MI/R-LIPUS_2.5_ (SI/R-LIPUS_2.5_) group compared to MI/R-sham (SI/R-sham) and MI/R-LIPUS_0.1_ (SI/R-LIPUS_0.1_) group.

### 3.5 LIPUS of 0.5 W/cm^2^ exerts cardioprotective effects by downregulating ASK1/JNK pathway under the hypoxic conditions

**Figure 7:**
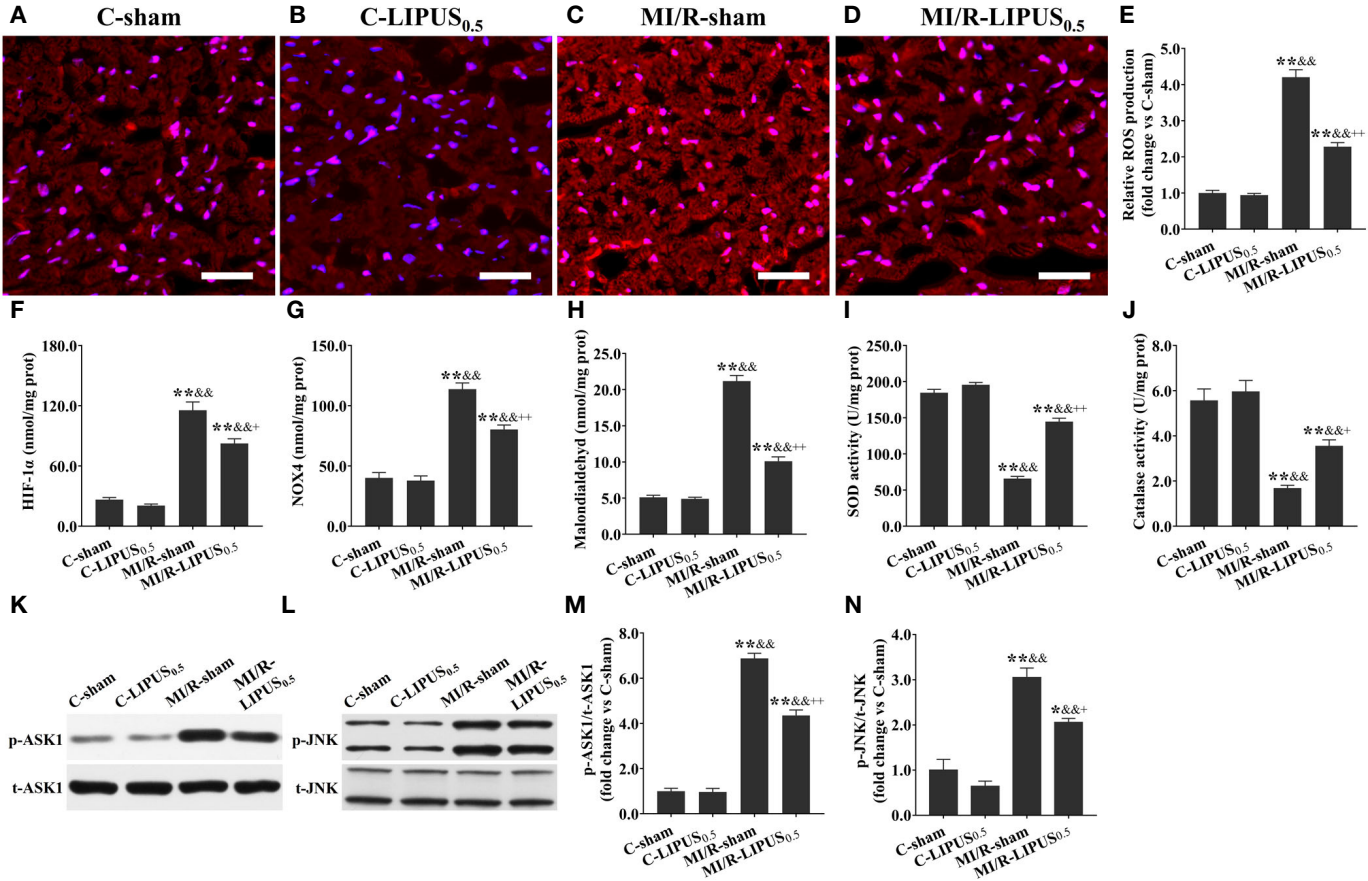
Low-intensity pulsed ultrasound (LIPUS) of 0.5 W/cm^2^ exerts cardioprotective effects by inhibiting the apoptosis signal-regulating kinase 1 (ASK1)/c-Jun N-terminal Kinase (JNK) pathway in the cardiomyocytes of the rats with myocardial ischemia/reperfusion (MI/R) injury. Representative images for reactive oxygen species (ROS) generation determined by dihydroethidium staining for the cardiomyocytes within the ischemic border zone in the rats **(A-D)**. Quantitative analysis for the effects of LIPUS on the levels of ROS **(E)**, hypoxia-inducible factor-1alpha (HIF-1α) **(F)**, NADPH oxidase 4 (Nox4) **(G)**, malondialdehyde (MDA) **(H)**, and on the activity of superoxide dismutase (SOD) **(I)** and catalase (CAT) **(J)** for the cardiomyocytes within the ischemic border zone in the rats. Representative western blots for phospho-ASK1 (p-ASK1), total ASK1 (t-ASK1), phospho-JNK (p-JNK), and total JNK (t-JNK) for the cardiomyocytes within the ischemic border zone in the rats **(K, L)**. Quantitative analysis for the effects of LIPUS on the ratios of p-ASK1/t-ASK1 and p-JNK/t-JNK for the cardiomyocytes within the ischemic border zone **(M, N)**. Magnification: 400×. Scale bar = 100 μm. All data are expressed as the mean ± SEM, N = 6 for each group. *: vs. C-sham group, P < 0.05; **: vs. C-sham group, P < 0.01; &: vs. C-LIPUS_0.5_, P < 0.05; &&: vs. C-LIPUS_0.5_, P < 0.01; +: vs. MI/R-sham, P < 0.05; ++: vs. MI/R-sham, P < 0.01.

**Figure 8:**
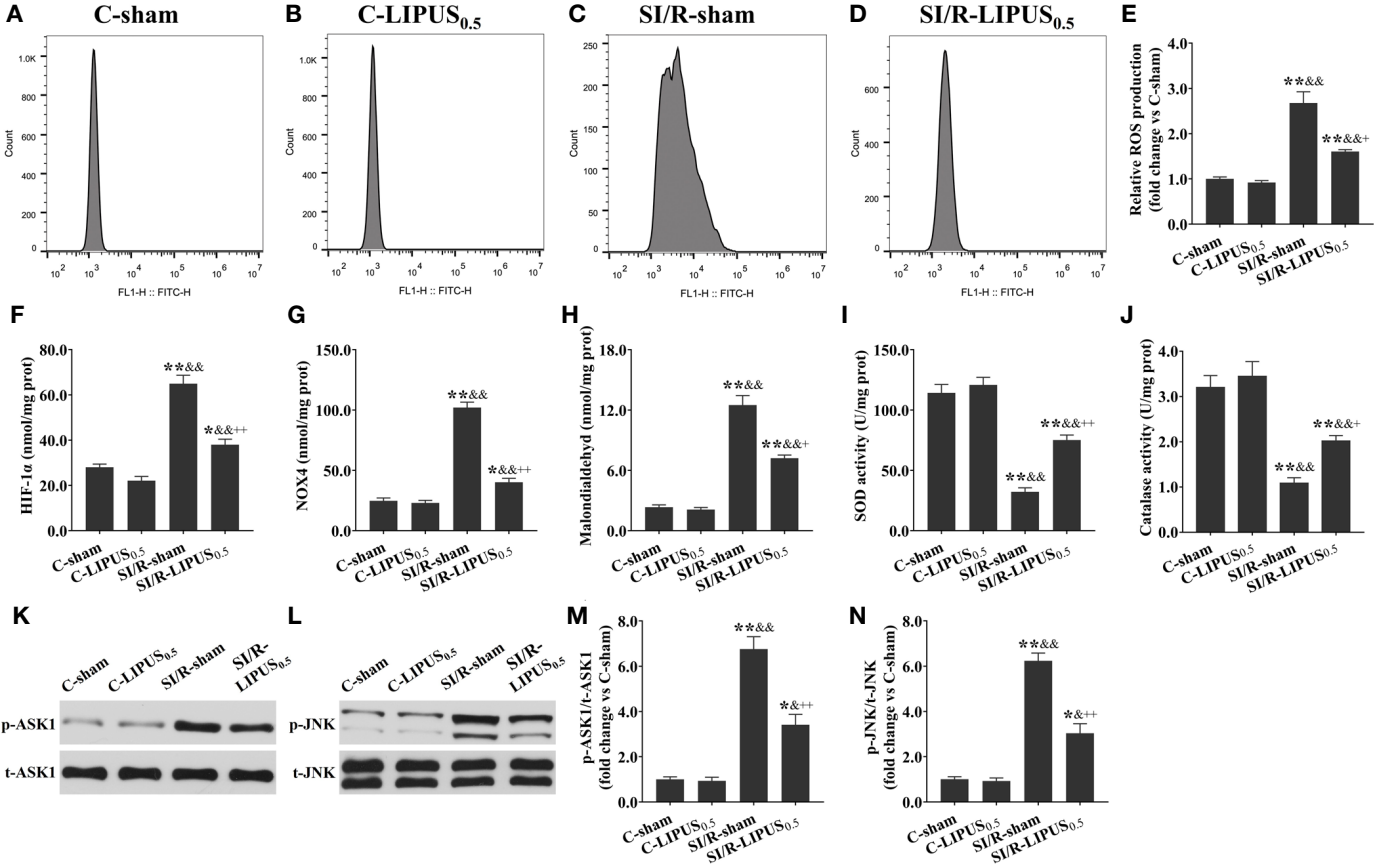
Low-intensity pulsed ultrasound (LIPUS) of 0.5 W/cm^2^ exerts cardioprotective effects by inhibiting the apoptosis signal-regulating kinase 1 (ASK1)/c-Jun N-terminal Kinase (JNK) pathway in the human pluripotent stem cell-derived cardiomyocytes (hPSC-CMs) with simulated ischemia/reperfusion (SI/R) injury. Representative images for reactive oxygen species (ROS) generation measured by flow cytometry analysis for the hPSC-CMs **(A-D)**. Quantitative analysis for the effects of LIPUS on the levels of ROS **(E)**, hypoxia-inducible factor-1alpha (HIF-1α) **(F)**, NADPH oxidase 4 (Nox4) **(G)**, malondialdehyde (MDA) **(H)**, and on the activity of superoxide dismutase (SOD) **(I)** and catalase (CAT) **(J)** for the hPSC-CMs. Representative western blots for phospho-ASK1 (p-ASK1), total ASK1 (t-ASK1), phospho-JNK (p-JNK), and total JNK (t-JNK) for the hPSC-CMs **(K, L)**. Quantitative analysis for the effects of LIPUS on the ratios of p-ASK1/t-ASK1 and p-JNK/t-JNK for the hPSC-CMs **(M, N)**. Magnification: 400×. Scale bar = 100 μm. All data are expressed as the mean ± SEM, N = 6 for each group. *: vs. C-sham group, P < 0.05; **: vs. C-sham group, P < 0.01; &: vs. C-LIPUS_0.5_, P < 0.05; &&: vs. C-LIPUS_0.5_, P < 0.01; +: vs. SI/R-sham, P < 0.05; ++: vs. SI/R-sham, P < 0.01.

Both *in vivo* and *in vitro*, the levels of ROS (**Figures 7A–E**, **8A–E**), HIF-1a (**Figures 7F**, **8F**), NOX4 (**Figures 7G**, **8G**), and MDA (**Figures 7H**, **8H**), the activity of SOD (**Figures 7I**, **8I**) and CAT (**Figures 7J**, **8J**), and the phosphorylation of ASK1 (**Figures 7K, M**, **8K, M**) and JNK (**Figures 7L, N**, **8L, N**) in cardiomyocytes were not significantly different between the C-sham and C-LIPUS_0.5_ group; The levels of ROS, HIF-1a, NOX4, and MDA were significantly increased while the activity of SOD and CAT were was apparently decreased in MI/R-sham (SI/R-sham) group compared to C-sham and C-LIPUS_0.5_ group accordingly; The dysregulation of ROS, HIF-1a, NOX4, MDA, SOD, CAT, ASK1, and JNK was partly alleviated in MI/R-LIPUS_0.5_ (SI/R-LIPUS_0.5_) group compared to the C-sham and MI/R-sham (SI/R-sham) group.

## 4 Discussion

### 4.1 Different intensities of LIPUS differently affects the injury and death of cardiomyocytes under the normoxic and hypoxic conditions

The medical application of ultrasound in diagnostic imaging has developed rapidly in the past few decades. Up to now, echocardiography has nearly provided a complete diagnosis of heart anatomy and cardiac performance (32). Ultrasound waves were found to be able to transmit through body tissues and induce molecular vibrations and collisions in recent years (33). LIPUS has been proven cardioprotective in several cardiovascular diseases, but rarely on MI/R injury (34, 35). A previous study has demonstrated that LIPUS of 0.5 W/cm^2^ attenuates cardiac inflammation of coxsackievirus B3 infection-induced viral myocarditis via regulating the mitogen-activated protein kinases family members (26). Based on the previous study, we designed this pilot study to clarify whether LIPUS with different intensities (0.1, 0.5, 2.5 W/cm^2^) exerts different effects on MI/R injury and tried to confirm which intensity of the LIPUS might be more suitable for the amelioration of MI/R injury. In our study, both *in vivo* and *in vitro*, LIPUS of 0.1 W/cm^2^ (LIPUS_0.1_) and 0.5 W/cm^2^ (LIPUS_0.5_) exert no significant effects on cardiomyocyte injury and death under the normoxic condition. In comparison, LIPUS_0.5_ exerts cardioprotective effects on the cardiomyocytes under hypoxic conditions. LIPUS of 2.5 W/cm^2^ (LIPUS_2.5_) aggravates cardiomyocyte injury and death both under normoxic and hypoxic conditions. These results demonstrate that the effects of LIPUS on cardiomyocytes are different from the cell conditions (normoxic condition or hypoxic condition) and the intensities of LIPUS.

### 4.2 Different intensities of LIPUS exert different effects on hypoxic responses under the normoxic and hypoxic conditions

**Figure 9:**
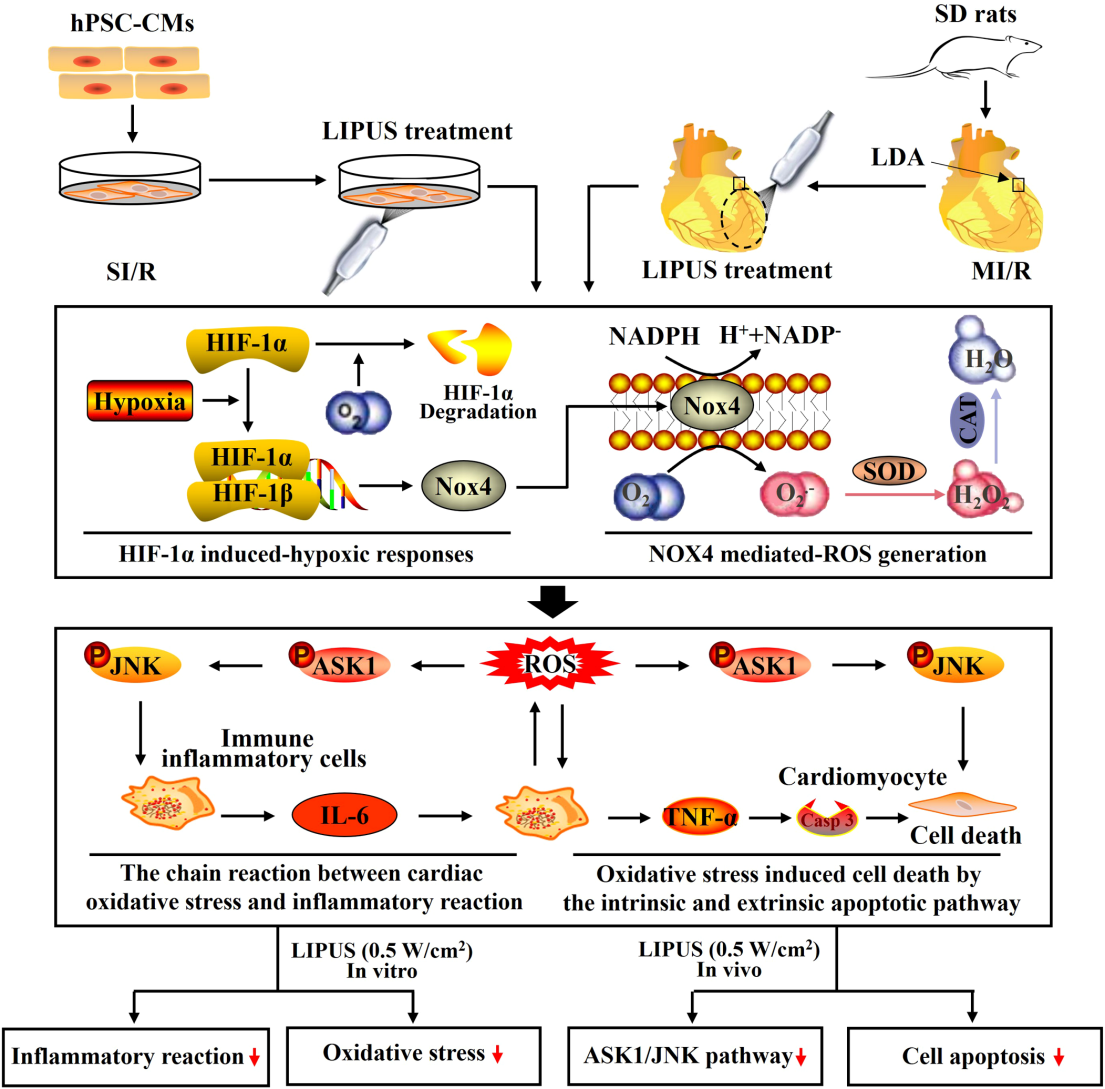
Schematic depicting the underlying mechanisms by which low-intensity pulsed ultrasound of 0.5 W/cm^2^ protects against myocardial ischemia/reperfusion (MI/R) injury. With heterodimers comprised of the hypoxia-inducible factor-1α (HIF-1α) subunit and HIF-1β subunit, hypoxia-inducible factors coordinate cellular adaptations to reduced O_2_ availability by regulating transcriptional programs in metabolism, angiogenesis, and erythropoiesis. Hypoxia increases and stabilizes HIF-1α by reducing prolyl hydroxylation and increasing the dimerization of HIF-1α and HIF-1β. HIF-1α is quickly degraded with a sufficient supply of O_2_. The O_2_-mediated degradation of HIF-1α is inhibited because of the hypoxic condition suffering from MI/R. Accumulated HIF-1α upregulates NADPH oxidase 4 (Nox4) by binding to a core DNA sequence in the gene of NOX4, upregulating NOX4 and inducing the overproduction of reactive oxygen species (ROS). ROS, including O_2_.- and H_2_O_2_, are mainly hydrolyzed by superoxide dismutase (SOD) and catalase (CAT). The increased ROS stimulates cardiomyocytes to release inflammatory cytokines by ROS signaling. The released inflammatory cytokines, including interleukin-6 (IL-6) and tumor necrosis factor-α (TNF-α), further stimulated cardiomyocytes to generate ROS by upregulating Nox4 in cardiomyocytes, forming a chain reaction between the cardiac oxidative stress and inflammatory reaction in the cardiomyocytes with MI/R injury. Under oxidative stress, ROS induces cell death by upregulating an intrinsic apoptotic pathway in which ASK1 and JNK are activated within cardiomyocytes. In addition, the released TNF-α also promotes cell death by activating an extrinsic apoptotic pathway in which the receptor of TNF-α is activated on the cytomembrane of cardiomyocytes and subsequently increases the expression of Casp-3, a major executioner of programmed cell death. Both *in vivo* and *in vitro*, LIPUS of 0.5 W/cm^2^ alleviates the MI/R jury, cardiac inflammatory reaction, and cardiac oxidative stress while LIPUS of 2.5 W/cm^2^ aggravates the MI/R jury, cardiac inflammatory reaction, and cardiac oxidative stress. The activation of the ASK1/JNK pathway is inhibited by LIPUS of 2.5 W/cm^2^ in cardiomyocytes both *in vivo* and *in vitro*.

With heterodimers comprised of the HIF-1α subunit and HIF-1β subunit, hypoxia-inducible factors coordinate cellular adaptations to reduced O_2_ availability by regulating transcriptional programs in metabolism, angiogenesis, and erythropoiesis. Hypoxia increases and stabilizes HIF-1α by reducing prolyl hydroxylation and increasing the dimerization of HIF-1α and HIF-1β (36, 37). HIF-1α is sensitive to O_2_ and is rapidly degraded under normoxic conditions (38). The O_2_-mediated HIF-1α degradation is inhibited in the cardiomyocytes suffering from MI/R. Increased HIF-1α is proven to aggravate MI/R injury by deteriorating oxidative stress and promoting inflammatory reactions (39). For instance, increased HIF-1α can upregulate Nox4 by binding to a core DNA sequence in the gene of NOX4, as well as increase the expression of TNF-α by activating macrophages in the myocardium (**Figure 9**) (40, 41). NOX4 is widely expressed in cardiomyocytes and is mainly responsible for excessive oxidative stress damage (42, 43). We noticed that the alterations of the NOX4 expression were not entirely consistent with that of HIF-1α. It indicated that NOX4 might not just be regulated by HIF-1α. Recent studies have demonstrated that the released inflammatory cytokines, such as TNF-α, can aggravate oxidative stress by upregulating Nox4 with complicated signal transduction pathways involved (42, 44). This point may be further supported by protein–protein networks among NOX4, TNF-α, IL-6, SOD, CAD, and so on.

### 4.3 Different intensities of LIPUS differently affects cardiac oxidative stress under the normoxic and hypoxic conditions

MI/R injury mainly results from abrupt metabolic and biochemical alterations within the cardiomyocytes. The burst generation of ROS is the primary initiator of cardiac inflammation and apoptosis during MI/R injury (**Figure 9**) (45, 46). Therefore, we paid close attention to the effects of LIPUS on cardiac oxidative stress in our study. It is well known that the increased production of ROS and MDA contributes to oxidative stress in cardiomyocytes with MI/R injury (47, 48). Hydrolases, such as SOD and CAT, are mainly responsible for the elimination of ROS. A decrease in the activity of the SOD and CAT deteriorates the oxidative stress by reducing the catalytic process of ROS under oxidative stress (**Figure 9**) (46, 48, 49). In our study, both *in vivo* and *in vitro*, LIPUS_0.1_ and LIPUS_0.5_ did not significantly affect the cardiac oxidative stress under the normoxic condition while LIPUS_0.5_ alleviated the cardiac oxidative stress obviously under the hypoxic condition. However, LIPUS_2.5_ deteriorated cardiac oxidative stress both under normoxic and hypoxic conditions. The different effects of LIPUS on cardiac oxidative stress correspond to its effects on MI/R injury and cardiac inflammation. Recent researches indicated a vital link between the downstream redox-sensitive signaling cascades and mechanosensing in the cardiovascular system. This link helps to transduce and transmit the physical force into a physiological response via ion channels, adhesion molecules, membrane receptor kinases, and other cellular components in the cardiovascular system (50, 51). With the oscillation of the mechanical waves, LIPUS is known to induce mechanical shear stress on tissues (52, 53). LIPUS_0.5_ may protect against MI/R injury through the downstream redox-sensitive signaling cascades and mechanosensing in the cardiovascular system. These points are worth further clarification.

### 4.4 Different intensities of LIPUS exert different effects on cardiac inflammation under the normoxic and hypoxic conditions

It is well demonstrated that hypoxia impairs the integrity of cardiomyocytes, and then cell death programs are activated (54, 55). Restoration of oxygen will further aggravate cardiomyocyte injury by inducing the overproduction of ROS, which subsequently activates the ROS pathway (43, 56). The damaged cardiomyocytes transmit oxidative stress signals to the nearby immune inflammatory cells and cardiomyocytes to release a cascade of inflammatory cytokines and chemokines, including IL-6, TNF-α, and MCP-1 (46, 57, 58). In turn, the released inflammatory cytokines and chemokines stimulated surviving immune inflammatory cells and cardiomyocytes to further aggravate oxidative stress, forming a chain reaction between cardiac inflammatory reaction and oxidative stress, ultimately resulting in cardiomyocyte apoptosis, a critical pathological outcome that predicts poor prognosis (**Figure 9**) (45, 59, 60). This may account for the phenomenon that cardiac inflammation and oxidative stress are progressive in most heart diseases. In our study, both *in vivo* and *in vitro*, LIPUS_0.1_ and LIPUS_0.5_ did not significantly affect the cardiac inflammation under the normoxic condition while LIPUS_0.5_ alleviated the cardiac inflammation apparently under the hypoxic condition. LIPUS_2.5_ deteriorated cardiac inflammation both under normoxic and hypoxic conditions. The different effects of LIPUS on cardiac inflammation, as well as on the injury and death of cardiomyocytes, coincide with the different effects of LIPUS on cardiac oxidative stress.

It is interesting that the levels of MCP-1 in the myocardium of rats were significantly increased in rats with ischemia/reperfusion and apparently decreased when the rats with ischemia/reperfusion were treated by LIPUS. However, neither ischemia/reperfusion nor LIPUS had a significant influence on the levels of MCP-1 in the culture medium of the hPSC-CMs. MCP-1, also known as C-C motif ligand 2, is an integral chemotactic factor primarily secreted by immune cells. MCP-1 can recruit monocytes and macrophages into the injured tissues to further promote immunoinflammatory response (61, 62). Immune cells in plasma that can secrete MCP-1 would be recruited into the injured myocardium in response to the inflammatory reaction and oxidative stress *in vivo* but not *in vitro*. This may be the reason that the effects of ischemia/reperfusion and LIPUS on the levels of MCP-1 are different between *in vivo* and *in vitro*.

### 4.5 LIPUS of 0.5 W/cm^2^ exerts cardioprotective effects by downregulating ASK1/JNK pathway under the hypoxic conditions

MI/R injury involves intricate signalings, including ROS signaling, which is particularly over-activated during reperfusion (43, 46, 56). LIPUS has been demonstrated to reduce cell injury by protecting against oxidative stress in several cardiovascular diseases (20–22). Therefore, we further attempt to clarify whether LIPUS protects against MI/R injury by regulating ROS signaling. Recent studies have demonstrated that the stress-activated protein kinases are a group of mitogen-activated protein kinases that can be activated in response to a variety of cellular stresses and play critical roles in the pathology of inflammatory reaction and oxidative stress (63, 64). Classically identified as a stress-activated protein kinase, JNK is crucial in controlling cell survival and inflammatory reaction through transcription factor c-Jun with the redox-sensitive signaling and ASK1 involved (65, 66). Therefore, we focused on the effects of LIPUS on the ASK1/JNK pathway in this study.

Under oxidative stress, ROS induces cell death by upregulating an intrinsic apoptotic pathway in which ASK1 and JNK are activated within cardiomyocytes (67, 68). In addition, the released TNF-α also promotes cell death by activating an extrinsic apoptotic pathway in which the receptor of TNF-α is activated on the cytomembrane of cardiomyocytes and subsequently increases the expression of Casp-3, a major executioner of programmed cell death (**Figure 9**) (68). ASK1 is a unique kinase that is activated by oxidative stress, which in turn mediates the activation of JNK activation (69). Apart from inducing apoptosis, activation of the ASK1/JNK pathway also aggravates cardiac inflammation in the heart with MI/R injury (70). Inhibition of the ASK1/JNK pathway has been demonstrated to reduce cardiac inflammation, oxidative stress, and apoptosis in several cardiovascular diseases with the involvement of complicated protein-protein networks indicated in **Figure 3** (71, 72). In our study, the ASK1/JNK pathway is upregulated in the cardiomyocytes with MI/R injury both *in vivo* and *in vitro*. The activation of the ASK1/JNK pathway is partly rectified in the cardiomyocytes treated by LIPUS_0.5_ both *in vivo* and *in vitro*. The inhibitory effects of LIPUS_0.5_ on the ASK1/JNK pathway coincide with its protective effects on cardiac inflammation, oxidative stress, and apoptosis. Our study, for the first time, demonstrated that LIPUS of 0.5 W/cm^2^ significantly ameliorates MI/R injury by downregulating the ASK1/JNK pathway. It will be a great welfare for the patients with MI/R injuries.

## 5 Conclusion

Based on the results of this study, we draw three conclusions: 1) Different intensities of LIPUS differently affect cardiomyocyte injury and death under normoxic and hypoxic conditions; 2) Different intensities of LIPUS exert different effects on cardiac oxidative stress and inflammatory reactions under normoxic and hypoxic conditions; 3) LIPUS of 0.5 W/cm^2^ exerts cardioprotective effects by modulating ASK1/JNK pathway under the hypoxic conditions.

## Data availability statement

The original contributions presented in the study are included in the article/**Supplementary Material**. Further inquiries can be directed to the corresponding author.

## Ethics statement

The animal study was approved by Institutional Animal Care and Use Committee at Renmin Hospital, Wuhan University, China. The study was conducted in accordance with the local legislation and institutional requirements.

## Author contributions

All authors have participated in the study conception and design. Material preparation, data collection, and analysis were mainly performed by QC, LL, and YH. The first draft of the manuscript was written by QC and LL. All authors commented on previous versions of the manuscript. All authors contributed to the article and approved the submitted version.

## Supplementary material

Supplementary Video S1 Observation of the human pluripotent stem cell-derived cardiomyocytes in the C-sham group under a digital light microscopy.

Supplementary Video S2 Observation of the human pluripotent stem cell-derived cardiomyocytes in the C-LIPUS0.1 group under a digital light microscopy.

Supplementary Video S3 Observation of the human pluripotent stem cell-derived cardiomyocytes in the C-LIPUS0.5 group under a digital light microscopy.

Supplementary Video S4 Observation of the human pluripotent stem cell-derived cardiomyocytes in the C-LIPUS2.5 group under a digital light microscopy.

Supplementary Video S5 Observation of the human pluripotent stem cell-derived cardiomyocytes in the SI/R-sham group under a digital light microscopy.

Supplementary Video S6 Observation of the human pluripotent stem cell-derived cardiomyocytes in the SI/R-LIPUS0.1 group under a digital light microscopy.

Supplementary Video S7 Observation of the human pluripotent stem cell-derived cardiomyocytes in the SI/R-LIPUS0.5 group under a digital light microscopy.

Supplementary Video S8 Observation of the human pluripotent stem cell-derived cardiomyocytes in the SI/R-LIPUS2.5 group under digital light microscopy.
